# Ectopic adrenal gland in an adult inguinal hernial sac: A case report

**DOI:** 10.1016/j.ijscr.2020.05.047

**Published:** 2020-05-30

**Authors:** Mersad Alimoradi, Etienne El-Helou, Hassan Sabra, Rawan Azaki, Mayssaloun Khairallah, Nazem Matta

**Affiliations:** Mount Lebanon Hospital, Mount Lebanon, Lebanon

**Keywords:** EACT, ectopic adrenocortical tissue, Inguinal hernia, Ectopic adrenocortical tissue, Case report

## Abstract

**Introduction:**

A 37-year-old male patient operated for inguinal hernia repair was found to have ectopic adrenocortical tissue in the hernial sac.

**Case presentation:**

A 37-year-old man was admitted for bilateral inguinal hernia. An uneventful open repair was done, and the resected hernial sacs were sent to pathology. Histopathology reported the presence of adrenocortical tissue in the right inguinal hernial sac.

**Discussion:**

Ectopic adrenocortical tissue (EACT) in the groin region is not an unusual finding in children, however, it’s rarely reported in adult patients. Only 9 cases have been reported in English describing EACT in an adult’s inguinal hernia. The finding can be attributed to the close proximity of the developing gonads and adrenal cortex during embryogenesis, and subsequent mechanical translocation of adrenocortical tissue during testicular descent. Some theoretical clinical implications exist for this condition, including secondary hyperplasia after adrenalectomy, adrenal insufficiency in certain situations, and possible neoplastic transformation. Generally, it is recommended that surgeons resect ectopic adrenal glands when identified intra-operatively. However, actively searching for these glands has no known benefit and carries some surgical risks, and is hence not recommended. It is reasonable as well, that clinicians keep the clinical implications of this finding in mind during future follow-ups with such patients.

**Conclusion:**

The presence of ectopic adrenocortical tissue in inguinal hernia sacs is a rare encounter in adults. The condition can have several theoretical clinical implications that need to be considered by surgeons while assessing patients in whom this phenomenon is observed.

## Highlights

• Ectopic adrenocortical tissue is a rare finding that can be encountered in inguinal hernial sacs of adults.
• Surgeons should be aware of this and are encouraged to resect Ectopic adrenocortical tissue when grossly identified.
• Secondary hyperplasia after adrenalectomy, adrenal insufficiency, and neoplastic transformation should all be considered.

## 1 Introduction

Ectopic adrenocortical tissue (EACT) is an unusual finding in adults and is more commonly encountered during groin surgeries in children [1]. The EACT usually regress after the first few years of life [2], however, in some rare cases like ours, it may persist well into adulthood. To the best of our knowledge, only 9 cases of EACT in an adult hernial sac were reported in the English literature, making it a rare finding [3,4]. The diagnosis is usually made retrospectively on histopathology. Several theoretical implications can be correlated with this finding, such as secondary hyperplasia occurring after adrenalectomy, adrenal insufficiency in certain patients, and the possibility of neoplastic transformation.

This case has been reported in line with the SCARE criteria [5].

## 2 Case description

A 37-year-old man, previously healthy, with no symptoms of endocrine dysfunction or any other systemic problems, presented for assessment of bilateral inguinal hernia. The hernias had been present for 1 year but had caused him no symptoms up until 1 month previously when he started feeling pain in the inguinal region after walking a certain distance. He was admitted for elective open bilateral inguinal hernia repair with mesh insertion. The surgery was performed in a routine fashion, adipose and fibromuscular tissue were grossly seen in the hernial sacs and were resected. We could not grossly identify any well-defined masses or nodules on either side. The resected tissue from both sides was sent to pathology as part of our hospital's protocol. The patient was discharged the next day with no complaints except for mild pain at surgical sites.

**Fig. 1:**
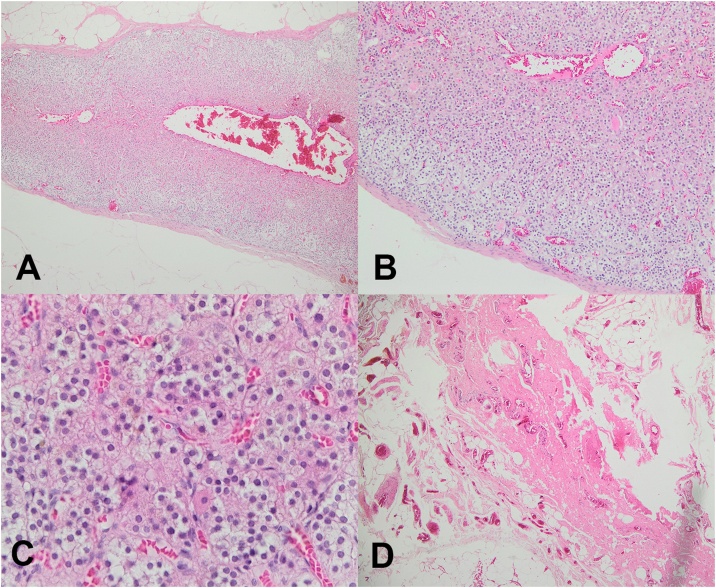
Pathology pictures of the resected tissue. Adrenocortical tissue enlarged 40 times (A), Adrenocortical tissue enlarged 100 times (B), Adrenocortical tissue enlarged 400 times (C), and Hernial sac (D).

One week later, the histopathology report came back reporting the presence of an ectopic adrenal gland in the tissue resected from the right inguinal hernia. The pathologist reported only adrenocortical tissue, yet no medullary tissue in the specimen. The pathology slides are shown in Fig. 1.

We followed up with the patient 2 months after the operation and he reported no new symptoms suspicious of endocrine dysfunction. No further testing was done.

## 3 Discussion

Ectopic adrenocortical tissue is defined as the presence of adrenocortical tissue outside of its normal location. This phenomenon was first described by Morgagni in 1740, who reported the presence of adrenocortical tissue in the vicinity of the spermatic cord of a child [6]. In fact, most of the cases of EACT described in the literature are in children [1], and only rarely in adults [7]. Some studies report that accessory adrenocortical tissue can be present in up to 50% of newborns, however, in most cases, this ectopic tissue does not persist past the first few years of life [2]. As for the location, EACT is reported in the celiac axis region (32%), broad ligament (23%), adnexa of the testis (7,5%), the spermatic cord (1–9,3%) the subcapsular upper pole of the kidney (0,1–6%) [1,8], and in individual reports in the placenta, liver, lung, and even the intracranial cavity [2,9]. In the adult groin region specifically, the most common EACT locations reported are within inguinal hernia sacs and along the spermatic cord, predominantly on the right side, as in our case [3]. The ectopic tissue in these cases usually goes unnoticed intraoperatively (86% of cases), and the diagnosis is made later by histopathology [3]. This could be explained by the similar appearance that these ectopic glands share with the surrounding adipose tissue encountered during groin surgery, where they’re usually described as small yellow nodules with an adipose-like aspect [3]. To the best of our knowledge, there are only 9 cases in the English literature reporting adrenocortical tissue in adult hernial sacs [3,4].

Considering the embryology of the adrenal cortex, and its proximity to the developing gonads, it is not very surprising that ectopic cortical tissue is occasionally encountered along the path of testicular descent. The adrenal cortex develops during the 4th and 5th weeks of gestation from mesothelial cells located between the mesentery root and the developing gonads. Given this close anatomical proximity between the developing gonads and the cortex, it is thought that the cortical tissue can be translocated mechanically during the descent of the gonads during embryogenesis [3]. The medulla, on the other hand, develops from the ectoderm of the neural crest and invades the developing cortex to become the definite medulla later during embryogenesis. This time and space relationship between the developing gonads, cortex, and medulla can explain why all of the reported cases of EACT contained only cortical, and yet never medullary tissue.

Other unusual findings reported in inguinal hernial sacs include the testes [10], ovaries [11,12], fallopian tubes [11,12], uterus [12], urinary bladder [11], ectopic appendix [11,13], epiploic appendagitis [14,15], glandular inclusions [16,17], endometriosis [18], parasitic granuloma [19], metastatic carcinomas [20], and sarcomas [21].

The presence of EACT in the groin region can have several clinical implications. After primary adrenalectomy for Cushing’s syndrome, secondary hyperplasia of the ectopic adrenocortical tissue can be responsible for the recurrence of the disease. Another implication should be considered in cases where the ectopic gland is the only adrenal tissue in the patient, or when it’s hyperfunctioning, causing regression of the normal adrenals. In such cases, the patient can theoretically develop adrenal insufficiency when the ectopic tissue is resected. A third implication is the hypothetical possibility of neoplastic transformation in ectopic adrenocortical tissue. It needs to be noted, however, that no cases describing adrenal insufficiency after the excision of ectopic adrenocortical tissue are present in the literature, nor are there reports describing neoplastic transformation in cases where the tissue persisted. Therefore, these implications remain theoretical. Based on this, surgeons are generally advised to resect lesions suspicious of ectopic adrenocortical tissue during groin surgery. However, routine active search for ectopic glands during these surgeries is not advised due to the risk of vascular injury associated with the dissection of the spermatic cord.

## 4 Conclusion

Ectopic adrenocortical tissue is one of the rare findings that can be encountered in inguinal hernial sacs of adults. Surgeons should be aware of this possibility during inguinal hernia repair and other groin surgeries and are encouraged to resect EACT when grossly identified. Nevertheless, actively searching for the gland is not recommended. This finding can have several theoretical clinical implications, including secondary hyperplasia after adrenalectomy, adrenal insufficiency in select patients, and lastly, the possibility of neoplastic transformation. Therefore, it would be reasonable to keep the clinical implications suggested in this article in mind during future follow-ups when such findings are encountered.

## Declaration of Competing Interest

This article has no conflict of interest with any parties.

## Sources of funding

This research did not receive any specific grant from funding agencies in the public, commercial, or not-for-profit sectors.

## Ethical approval

The study type is exempt from ethical approval.

## Consent

Written informed consent was obtained from the patient for publication of this case report and accompanying images. A copy of the written consent is available for review by the Editor-in-Chief of this journal on request.

## Author contribution

Writing the paper: Mersad Alimoradi, Hasan Sabra.

Data collection: Etienne El-Helou, Maysaloun Khairallah, Rawan Azaki.

Supervision: Nazem Matta.

## Registration of research studies

Name of the registry: N/A.

Unique identifying number or registration ID: N/A.

Hyperlink to your specific registration (must be publicly accessible and will be checked): N/A.

## Guarantor

Dr. Nazem Matta.

## Provenance and peer review

Editorially reviewed, not externally peer-reviewed.
